# Megapneumonia Coinfection: *pneumococcus, Mycoplasma pneumoniae,* and *Metapneumovirus*

**DOI:** 10.1155/2012/310104

**Published:** 2012-10-17

**Authors:** Kam Lun Hon, Margaret Ip, Winnie Chiu Wing Chu, William Wong

**Affiliations:** ^1^Department of Paediatrics, The Chinese University of Hong Kong, 6/F Clinical Sciences Building, Prince of Wales Hospital, Shatin, Hong Kong; ^2^Department of Microbiology, The Chinese University of Hong Kong, Prince of Wales Hospital, Shatin, Hong Kong; ^3^Department of Imaging and Interventional Radiology, The Chinese University of Hong Kong, Prince of Wales Hospital, Shatin, Hong Kong

## Abstract

We report a young girl who died of *Streptococcus pneumoniae* 19A pneumonia, septic shock, and hemolytic uremic syndrome despite prior pneumococcal vaccination, appropriate antibiotics, and aggressive intensive care support. Serotype 19A is not covered by the 7- or 10-valent pneumococcal vaccines. *Mycoplasma pneumoniae* and *metapneumovirus* were simultaneously detected by PCR in the nasopharyngeal and tracheal aspirates. The *pneumococcus* is penicillin sensitive. Although infections with each of these pathogens alone are typically mild, this case highlights that co-infection with the triple respiratory pathogens possibly contributed to the fatal outcome of this child. Also, the new policy in Hong Kong to use PCV13 may help prevent further cases of serotype 19A infections.

## 1. Case

**Figure 1:**
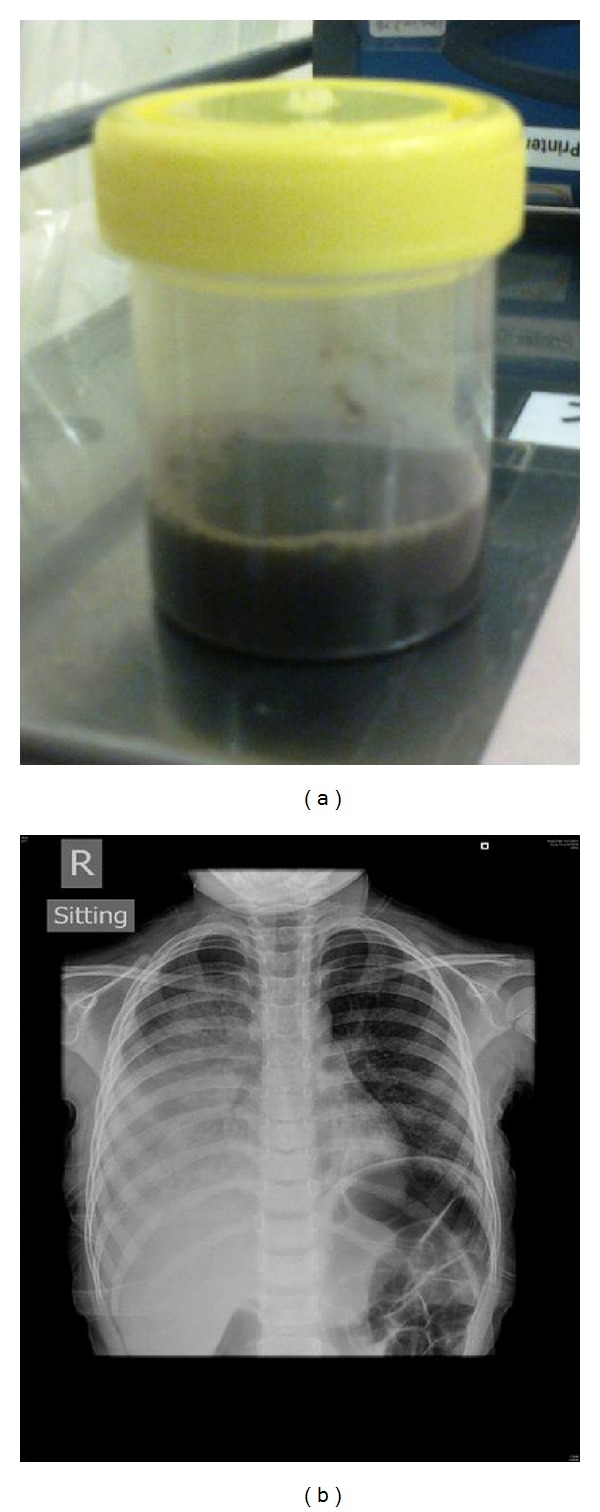
Right pleural effusion associated with pneumococcal pneumonia. *Mycoplasma* and *Metapneumovirus* were also detected in the tracheal aspirates to suggest co-infection in this case. The drained pleural fluid was dark and turbid and yielded *Streptococcus pneumoniae* (serotype 19A).

A previously healthy 4-year-old girl became dyspneic and critically ill with pneumonia and multiorgan system failure. She had been febrile with chills for 4 days (39°C), breathless and coughing for 2 days. She had visited the general practitioners twice, and treated symptomatically but with no antibiotic prescription. She had vomited and passed watery stool. Her immunizations were up-to-date and she received one dose of 7-valent pneumococcal vaccine at age 2 years. The young brother had recovered from a recent febrile coughing episode. Because of increasing shortness of breath, the mother took her to the emergency department. Her vitals were: heart rate 165/min, respiratory rate 30/min, tympanic temperature 36.7°C, SaO_2_ 79% in room air, and decreased air entry to right lung. Supplemental oxygen of 6 liter/min was immediately given. Chest radiography showed right sided pneumonia with pleural effusion (Figure 1).

On admission to the pediatric ward, temperature was 36.9°C, pulse 125/min, respiratory rate 46/min, blood pressure 94/52 mmHg, and SaO_2_ 95% with 100% oxygen. Shortly, the patient became lethargic with cold peripheries, cyanosis, and insucking chest. She was given normal saline boluses and dobutamine (10 mcg/kg/min) and promptly transferred to the pediatric intensive care unit (PICU). There, she received cardiopulmonary resuscitation (13 minutes) because of cardiopulmonary failure, septic shock, and bradycardia. She was ventilated and treated with intravenous cefotaxime, high dose ampicillin, vancomycin, clarithromycin, packed cell, fresh frozen plasma, and cryoprecipitate transfusions. Abnormal laboratory findings included hemoglobulin 8.0 g/dL, platelets 87 × 10^9^/L, and white cell count 1 × 10^9^/L; activated partial thromboplastin time APTT was 47.9 seconds, prothrombin time PT 13.4 seconds, d-dimer 2192 ng/mL, C-reactive protein 51.9 mg/L, plasma urea 18.2 mmol/L, creatinine 214 umol/L. Her lowest PaO_2_/FiO_2_ was 42, and arterio-alveolar gradient 610 mmHg. Echocardiography showed reduced cardiac contractility (ejection fraction 48%) but no pericardial effusion. The pleural effusion was drained, and 40 mL of dark turbid fluid yielded drained (Figure 1 insert). Despite multiple inotrope infusions of dobutamine, adrenaline, noradrenaline, and milrinone, her mean blood pressure remained low (50 mmHg) and she suffered another episode of cardiac arrest 9 hours following admission. Hemodiafiltration was instituted for intractable septic shock, renal failure, acidosis, and fluid retention. The patient ran a deteriorating course despite aggressive ICU support. She developed another episode of cardiac arrest and succumbed 39 hours following hospitalization.

Nasopharyngeal and tracheal aspirates yielded *Mycoplasma pneumoniae* (by polymerase chain reaction PCR) and human *Metapneumovirus* RNA (by RT-PCR). Blood culture, pleural fluid, and tracheal aspirate yielded leucocytes and *Streptococcus pneumoniae *(serotype 19A) sensitive to cefotaxime and penicillin. CSF was not obtained during the critical episode.

Postmortem examination confirmed generalized necrotizing pneumonia with diffuse alveolar damage and right middle lobe hemorrhagic infarct. The proximal tubules of both kidneys showed acute tubular necrosis but no microangiopathic thrombosis. Histology of the heart and the brain was unremarkable. Tissue cultures were negative.

## 2. Discussion

*Pneumococcus* is an important pathogen in childhood [1–3]. Invasive pneumococcal disease refers to unlocalised bacteremia, pneumonia, or meningitis. Despite the availability of effective vaccines, new serotypes continue to evolve [1–3]. The Hong Kong Government introduced the 7-valent polysaccharide vaccine in September 2009. In 2010, the vaccine was changed to the 10-valent vaccine and in 2011 recommendation was made to switch to a 13-valent vaccine. Locally, the coverage of the 7-valent or 10-valent and 13-valent vaccines was 65% and 90%, respectively [2]. The parents reported that the child received one prior dose of 7-valent vaccine before 3 years of age in early 2010. It is possible that 19A was a commonly circulating strain before the introduction of the 10-valent or 13-valent vaccines. The 13-valent vaccine should stop circulation of 19A when the program is fully implemented. 

History of immunization with the 7-valent vaccine is not a guaranteed prevention against pneumococcal infection in children [1–3]. Evolving serotypes associated with severe lobar pneumonia, pleural effusion, and PICU admission despite prior immunization have been previously reported locally [4]. 

Evidenced-based guidelines for management of infants and children with community-acquired pneumonia (CAP) were prepared by an expert panel comprising clinicians and investigators representing community pediatrics, public health, and the pediatric specialties of critical care, emergency medicine, hospital medicine, infectious diseases, pulmonology, and surgery [5]. Amoxicillin should be used as first-line therapy for previously healthy, appropriately immunized infants and preschool-aged children with mild to moderate CAP suspected to be of bacterial origin. Amoxicillin provides appropriate coverage for *S. pneumoniae*, the most prominent invasive bacterial pathogen. Macrolide antibiotics should be prescribed for treatment of children (primarily school-aged children and adolescents) evaluated in an outpatient setting with findings compatible with CAP caused by atypical pathogens. Laboratory testing for *M. pneumoniae* should be performed if available in a clinically relevant time frame. Ampicillin or penicillin G should be administered to the fully immunized infant or school-aged child admitted to a hospital ward with CAP when local epidemiologic data document lack of substantial high-level penicillin resistance for invasive *S. pneumoniae*. Empiric therapy with a third-generation parenteral cephalosporin (ceftriaxone or cefotaxime) should be prescribed for hospitalized infants and children who are not fully immunized, in regions where local epidemiology of invasive pneumococcal strains documents high-level penicillin resistance, or for infants and children with life-threatening infection, including empyema. Empiric combination therapy with a macrolide (oral or parenteral), in addition to a beta-lactam antibiotic, should be prescribed for the hospitalized child for whom *M. pneumonia* and *C. pneumoniae* are significant considerations; diagnostic testing should be performed if available in a clinically relevant time frame. Accordingly, earlier initiation of antibiotics might have increased the chances of survival in this child. 

Antibiotic resistance has also developed in Hong Kong [1, 2]. The serotype 19A is especially virulent and may be difficult to isolate in patients who have already been started on antibiotics. The pathogen has been reported to be associated with the hemolytic uremic syndrome [6–11]. Penicillin can be used in sensitive *pneumococcus* [1, 2]. In patient with pneumonia not responding satisfactory initially, more invasive investigative/therapeutic management including a pleural drain for biologic specimen is indicated to guide management. Local antimicrobial sensitivity in PICU patients has been reported [1, 2]. The pathogen was sensitive to penicillin and cefotaxime in this case. In patients not responding satisfactorily with initial antibiotics but with known penicillin sensitivity, a higher dose of penicillin should be tried.

Coinfections by viral and bacterial agents in critically ill patients have been reported [12–15]. *Mycoplasma* pneumoniae usually affects older children and a clinical entity of atypical pneumonia or “walking” pneumonia. *Metapneumovirus* usually causes co-infection [13]. Occasionally, both pathogens can cause severe acute respiratory symptoms just like SARS (severe acute respiratory syndrome) [16]. 

In-house real-time RT-PCRs were performed according to hospital laboratory standard operating procedures for the qualitative detection of human *Metapneumovirus* (hMPV) RNA and of *Mycoplasma pneumoniae* (MP). The target of amplification for hMPV was the nucleoprotein gene (N gene) with primer sequences and method as described by Hopkins et al. and 45 cycles were run on real-time PCR (ABI prism 7900 HT FAST) [17]. Positive and negative controls were included, and a Ct value of ≤37 was considered positive. The target of amplification for MP was the ADP-ribosylating toxin gene encoding the CARDS (community-acquired respiratory distress syndrome) toxin using primer pairs and method as described by Winchell et al. [18]. Both internal DNA control and a positive and negative control were included in the reaction run. A Ct value ≤34 was considered positive.

It is difficult to ascertain if *Metapneumovirus* and *Mycoplasma* had contributed to this fatal illness. These pathogens were detected in the tracheal and the nasopharyngeal aspirates but not in the postmortem lung tissue cultures. Unlike the nasopharynx, the presence of any pathogens in the tracheal aspirates represents infection in the lower respiratory tract rather than carriage in the upper airway. Both pathogens are known to cause pneumonia on their own and both are not commonly carried by healthy young persons [5, 13, 19–22]. These facts support the argument that they were copathogens with *pneumococcus* which was found in pleural fluid, tracheal aspirates, and blood. In conclusion, the simultaneous isolations of 3 respiratory viral and bacterial pathogens have not been reported in our locality and may contribute to the fatal outcome of this unfortunate child.
